# From Complexity to Competency: International Nursing Perspectives on Comprehensive Geriatric Assessment

**DOI:** 10.3390/geriatrics11030073

**Published:** 2026-06-17

**Authors:** Evangelos C. Fradelos, Sini Eloranta, Ioanna Tsatsou, Ioanna Dimitriadou, Susanna Mört, Nina Korsström, Anna Lundberg, Magdalena Häger, Jekaterina Šteinmiller, Agita Melbarde-Kelmere, Kristaps Circenis, Sigrun S. Skuladottir, Ingibjörg Hjaltadòttir, Maria Saridi

**Affiliations:** 1 Department of Nursing, University of Thessaly, 41500 Larissa, Greece; ioadimitriadou@uth.gr (I.D.); msaridi@uth.gr (M.S.); 2 Department of Nursing Science, University of Turku, 20014 Turku, Finland; sini.eloranta@turkuamk.fi; 3 Faculty of Health and Wellbeing, Turku University of Applied Sciences, 20520 Turku, Finland; susanna.mort@turkuamk.fi (S.M.); nina.korsstrom@turkuamk.fi (N.K.); 4 Department of Nursing, University of West Attica, 12243 Egaleo, Greece; 5 Department of Nursing, Åland University of Applied Sciences, 22100 Mariehamn, Finland; annasofia.lundberg@ha.ax (A.L.); magdalena.hager@ha.ax (M.H.); 6 Department of Nursing, Tallinn Health University of Applied Sciences, 13418 Tallinn, Estonia; jekaterina.steinmiller@ttk.ee; 7 Department of Nursing and Midwifery, Faculty of Health and Sport Sciences, Rīga Stradiņš University, LV-1007 Riga, Latvia; agita.melbarde-kelmere@rsu.lv (A.M.-K.); kristaps.circenis@rsu.lv (K.C.); 8 Faculty of Nursing and Midwifery, School of Health Sciences, University of Iceland, 102 Reykjavik, Iceland; sigrunsunna@hi.is (S.S.S.); ingihj@hi.is (I.H.)

**Keywords:** comprehensive geriatric assessment (CGA), primary health care (PHC), geriatric nursing, nursing education, qualitative research

## Abstract

**Background/Objectives:** Population aging has increased the demand for comprehensive and coordinated care for older adults, placing primary health care (PHC) nurses at the forefront of comprehensive geriatric assessment (CGA) implementation. However, limited evidence exists regarding nurses’ experiences and educational needs related to CGA across different European healthcare contexts. This study aimed to explore the experiences, perceived challenges, and educational needs of PHC nurses regarding the implementation of CGA in five European countries. **Methods:** A qualitative study design was employed using five focus groups, one in each participating country: Greece, Iceland, Finland, Estonia, and Latvia. A total of 29 PHC nurses participated in semi-structured interviews. Data were analyzed using reflexive thematic analysis. **Results:** Three overarching themes and nine subthemes were identified in relation to good practices in CGA: (1) embracing the complexity of holistic assessment; (2) balancing consistency with flexibility in clinical practice; and (3) advancing professional expertise through structured learning. Participants highlighted the importance of standardized assessment tools and effective multidisciplinary collaboration. At the same time, they reported significant gaps in formal education, limited organizational resources, and insufficient training in cultural competence, which hinder consistent CGA implementation. **Conclusions:** PHC nurses recognize the value of a holistic and structured approach to CGA but require clearer educational pathways and evidence-based training to address the complex needs of older adults. Strengthening curricula that emphasize interprofessional collaboration, practical competencies, and contextual adaptability may support more effective and sustainable implementation of CGA in primary care settings across Europe.

## 1. Introduction

Population aging is a global phenomenon that has led to a growing number of older adults requiring complex and long-term health care services [1]. Older age is commonly defined as 60 years and above and is associated with increased multimorbidity, functional decline, and heightened vulnerability, resulting in diverse and interrelated physical, psychological, and social care needs [2]. These needs include maintaining health and functional ability, ensuring safety, preserving autonomy, preventing social isolation, and supporting meaningful participation in society [3,4].

Primary health care (PHC) plays a central role in responding to these multidimensional needs, as it often represents the first and most continuous point of contact within the healthcare system [5]. Through ongoing monitoring, health promotion, and coordination of services, PHC is uniquely positioned to support older adults in maintaining independence and quality of life. Consequently, effective models of geriatric care within primary care settings are essential to address the growing complexity associated with population aging.

Geriatrics is the medical specialty focused on the prevention, diagnosis, and management of health conditions in older adults, while geriatric nursing represents a specialized area of practice aimed at delivering holistic, person-centered care that accounts for age-related physiological changes and complex care needs [6,7]. Nurses play a pivotal role in coordinating care for older people, particularly those with multimorbidity, functional limitations, and social vulnerability, and are often central to communication between patients, families, and multidisciplinary teams [8].

A cornerstone of high-quality geriatric care is the comprehensive geriatric assessment (CGA), a multidimensional and interdisciplinary process designed to evaluate an older person’s medical, functional, psychological, and social status in order to develop a coordinated and individualized care plan [9]. CGA involves systematic data collection using standardized assessment tools, clinical examination, medication review, and evaluation of functional and environmental factors. The process is conducted collaboratively by a multidisciplinary team—including physicians, nurses, therapists, and social workers—and actively involves the older person and their family in care planning and decision-making [10,11]. CGA can be implemented across care settings and is particularly valuable in primary care, where early identification of risks and proactive intervention can support aging in place and prevent avoidable functional decline [11].

Within the CGA process, nurses serve a central coordinating role by conducting comprehensive assessments, applying standardized instruments and patient-reported outcome measures, documenting findings, and facilitating communication among team members. Effective nursing involvement is essential to ensure continuity, person-centeredness, and integration of care across settings [12,13,14]. However, despite the recognized value of CGA, its implementation in PHC remains inconsistent.

Existing research on CGA has predominantly focused on hospital-based settings, while evidence addressing its use in PHC remains limited [9,15]. Moreover, there is a lack of standardized approaches and structured educational frameworks to support nurses in performing CGA consistently and competently across different healthcare contexts [9,14]. This gap may hinder effective implementation and reduce the potential benefits of CGA in everyday primary care practice.

Therefore, this study aimed to explore and analyze the experiences and educational needs of primary health care nurses regarding the implementation of comprehensive geriatric assessment in five European countries. By identifying perceived challenges, good practices, and training needs, the study seeks to inform the development of a targeted and realistic educational curriculum to support nurses in delivering high-quality, holistic geriatric care in primary care settings. Given the variations in nursing professional profiles in Europe, ranging from highly autonomous clinical coordinators to family health practitioners, and the underlying system differences across the surveyed nations, a cross-contextual structural analysis is provided to avoid informational bias and contextualize the qualitative findings.

## 2. Materials and Methods

### 2.1. Study Design and Setting

A qualitative study design using focus groups was employed. The reporting of the study followed the Consolidated Criteria for Reporting Qualitative Research (COREQ) checklist to ensure methodological rigor and transparency. The complete checklist is available as a Supplementary File.

Five focus groups were conducted, one in each participating country: Greece, Iceland, Finland, Estonia, and Latvia. The selection of these five specific European countries was determined by the structure of the international consortium funded under the European Commission’s Erasmus+ framework. Methodologically, this multi-country composition provides a highly diverse sample that spans different European geographic regions (Southern, Northern, Baltic, and Nordic Atlantic), varying PHC systems, and distinct nursing educational frameworks. This systemic and geographic variation allows for a comprehensive exploration of shared challenges, localized good practices, and universal educational gaps, thereby enhancing the cross-contextual relevance and transferability of the qualitative findings.

In total, 29 primary health care (PHC) nurses participated. The focus group consisted of six participants from Finland, coded sequentially from FIN_RN1 to FIN_RN6; five participants from Estonia, EST_RN1 to EST_RN5; and five participants from Latvia, coded from LT_RN1 to LT_RN5. Additionally, the sample featured six participants from Greece, coded from GR_RN1 to GR_RN6, and seven participants from Iceland, who were coded sequentially from ICL_RN1 to ICL_RN7.

Focus groups were selected to facilitate interaction among participants, allowing the exchange of professional experiences and the identification of shared challenges and educational needs related to CGA [16,17]. This approach supports the exploration of collective perspectives and enables a deeper understanding of practice-based knowledge relevant to curriculum development and workforce preparation [18].

### 2.2. Data Collection and Duration

Data were collected using a semi-structured interview guide consisting of 14 specific questions, which was utilized across all focus groups to ensure structural consistency while allowing flexibility to explore emerging topics [19]. Each session lasted between 92 and 117 min, providing sufficient time for in-depth discussion.

The focus group interview guide was collaboratively reviewed, pre-tested, and conceptually validated by the international consortium members to ensure cross-cultural adaptability and clarity before formal data collection. To capture the targeted training needs and practice experiences systematically, the focus group interview guide was structured around the following core questions:

1. How long have you been working as a nurse in primary health care/social and health care?
2. What has been your experience with older adults in your practice? Geriatric assessment?
3. Have you received education on how to carry out a comprehensive geriatric assessment, and what kind of education?
4. Could you describe what and how you understand by a comprehensive geriatric assessment? What experiences do you have with geriatric assessment?
5. How often do you have to use geriatric assessment in your work?
6. How do you perform the health evaluation of the elderly?
7. Describe the main areas that arise specifically in the nurse’s work in this geriatric assessment.
8. What tools do you use when you do a geriatric assessment? (e.g., work tools, colleague support, and mentors)
9. In your opinion, how effective is the geriatric assessment in improving the care of older adults?
10. What challenges do you think there could be in this geriatric assessment?
11. Can you describe a situation where you felt certain, successful, or uncertain about the matter?
12. What areas do you feel you need more education in regarding elderly assessment?
13. How do you currently update your knowledge and skills in caring in geriatric care?
14. What are your ideas for developing education (contents, methods, and evaluation) of comprehensive geriatric assessment?

The focus group interviews were conducted in the native language of each participating country (Finnish, Estonian, Latvian, Greek, and Icelandic) to facilitate natural expression and depth of discussion among participants. All sessions were audio-recorded and transcribed verbatim to ensure accurate data capture. To preserve linguistic and contextual accuracy, primary transcription was performed by local research team members fluent in the respective native languages. Subsequently, a secondary researcher within each local team independently cross-checked the transcripts against the original audio recordings to verify accuracy and resolve any discrepancies prior to data processing.

The preliminary thematic analysis and initial coding were then performed by these bilingual local researchers directly on the native-language transcripts, preventing premature loss of meaning or cultural nuance. Following this initial stage, the established codes, structural subthemes, and illustrative participant quotes were translated into English to allow for cross-country synthesis and publication. This translation process was executed by the bilingual co-authors from each respective country profile. To ensure rigorous cross-linguistic validity and conceptual equivalence, a back-translation procedure was applied to a representative subset of the data, and final consensus on the English clinical terminology was achieved through collaborative peer-debriefing sessions within the international research team.

### 2.3. Data Analysis

Data were analyzed using reflexive thematic analysis, a flexible qualitative method suitable for identifying patterns and meanings across participant accounts [20]. This approach enabled systematic exploration of nurses’ experiences, perceptions, and educational needs related to CGA in primary health care.

In alignment with the epistemological framework of reflexive thematic analysis [20], the research team recognized that themes were actively constructed through the intersection of the data and the researchers’ own professional subjectivities. The analysis was conducted by a multi-disciplinary, international team of nursing educators, clinical researchers, and public health experts representing five European nations. The team’s extensive background in PHC, geriatric care, and specialized nursing practices functioned as an interpretive resource, enabling a deeper, contextually grounded understanding of the structural, educational, and operational realities described by the participants. Throughout the coding and theme development phases, reflexive journals and peer-debriefing sessions were utilized to critically examine how our professional assumptions and regional healthcare experiences shaped data interpretation.

The analysis followed a six-step process based on established methodological guidance [20,21,22]:

1. Familiarization with the data through repeated reading of transcripts.
2. Initial coding to identify meaningful units and recurring patterns. To ensure analytical rigor, two coders independently coded a subset of the transcripts. Following this initial phase, the two coders met to compare their independently generated codes, discuss variations in interpretation, and achieve a consensus. A unified coding framework was then developed and applied across the entire dataset, with regular peer-debriefing sessions held throughout the process to maintain consistency.
3. Grouping codes into potential themes reflecting key concepts, such as competencies and educational needs.
4. Reviewing themes for internal coherence and consistency with the dataset.
5. Defining and naming themes to clarify their scope and meaning.
6. Synthesizing themes into a coherent analytical narrative.

This structured analytical process supported the identification of good practices and gaps in CGA-related education, providing an evidence-based foundation for the development of targeted and practice-oriented nursing curricula.

### 2.4. Measures Taken for Trustworthiness

To ensure the qualitative integrity, validity, and rigor of the findings across multiple international contexts, specific measures for trustworthiness were established based on the criteria of credibility, transferability, dependability, and confirmability.

To ensure the findings accurately reflect the participants’ clinical realities, a multi-coder process was used where two researchers independently coded a representative subset of transcripts, meeting subsequently to resolve variations and establish a unified coding framework through consensus. Verbatim participant quotes from all distinct national cohorts are presented within the findings to ground the analytical narrative directly in empirical evidence.

To allow readers to evaluate the applicability of these findings to other healthcare settings, the paper provides a transparent and detailed contextualization of the sample characteristics and details the varying national healthcare system architectures, nursing educational systems, and distinct operational boundaries of PHC in the participating countries.

Also, the research processes were fully standardized across the international consortium using an identical semi-structured interview guide and structured transcription/back-translation procedures. Independent secondary cross-checking of the native-language transcripts against the original audio files was conducted in each country to verify literal accuracy prior to synthesis.

To manage, minimize, and explicitly navigate potential researcher subjectivities and interpretative biases stemming from the authors’ diverse backgrounds in public health and nursing education, researchers maintained reflexive journals throughout the data analysis pipeline. Cross-country peer-debriefing sessions were regularly executed by the international research team to critique, refine, and collectively approve the structural derivation of final global themes and localized subthemes.

### 2.5. Ethical Considerations

This study was conducted in strict accordance with the ethical principles outlined in the Declaration of Helsinki. The study protocol was formally reviewed and approved by the Nursing Department Ethics Committee of the University of Thessaly (Approval No. 26/11 March 2024). Prior to data collection, all participants were provided with detailed oral and written information regarding the study’s aims, the voluntary nature of their participation, and their right to withdraw at any stage without any professional or personal repercussions.

Written informed consent was explicitly obtained from all subjects involved in the study. To ensure absolute anonymity and data confidentiality, all identifiable personal information was removed during the transcription process, and participants were assigned alphanumeric codes (e.g., FIN_RN1, GR_RN4) for data analysis and reporting. All digital audio recordings and transcripts were stored securely on password-protected institutional servers accessible only to the primary research team.

## 3. Results

### 3.1. Participants’ Characteristics

Prior to the focus group sessions, all participants completed a brief demographic questionnaire to record personal and professional data. A total of 29 PHC nurses participated in the five focus groups across the partner nations. The cohort was predominantly female (93.1%, *n* = 27), with male representation (6.9%, *n* = 2) occurring exclusively within the Greek sample. The participants represented a broad professional spectrum; clinical working experience ranged from 2.5 to 40 years, with a mean experience of 16.3 years. Educational backgrounds varied notably by region, with 31.0% (*n* = 9) holding a Master’s degree, 51.7% (*n* = 15) holding a Bachelor’s degree, and 17.2% (*n* = 5) possessing vocational or registered nurse diplomas. Participant age was not uniformly recorded across all cohorts and is explicitly denoted as not available. A comprehensive summary of these descriptive characteristics is presented in Table 1.

### 3.2. Thematic Analysis Results

The thematic analysis conducted revealed three major themes and nine subthemes regarding the concept of “Good Practices in Comprehensive Geriatric Assessment” (Table 2).

### 3.3. Theme 1. Embracing Complexity: A Holistic Framework for Comprehensive Assessment

This theme consisted of two subthemes: *(a) intersecting domains: physical, psychological, and social well-being, and (b) precision in practice: utilizing targeted methods for geriatric assessment*.

All the nurses addressed the value of the integrated geriatric assessment. For nurses, a comprehensive assessment focuses on the assessment of all parameters that affect the health of older people, such as physical, psychological, and social aspects. Through this approach, potential risks and problems are identified early, resulting in personalized care and a better quality of life. The holistic approach enhances prevention and support for the well-being of older people.

#### 3.3.1. Intersecting Domains: Physical, Psychological, and Social Well-Being

Nurses emphasized the dynamics and the potential of interaction between various health domains of a person. Thus, assessing physical health alongside psychological and social factors is crucial to developing a thorough understanding of elderly patients’ needs.

*“A comprehensive study that takes the elderly person into account holistically… physical, psychological, social, cognitive, individual.”*

(FIN_RN1)

*“It’s just physical and mental, social ability, and, and here, and resources, and, and relationships, and yes.”*

(ICL_RN1)

#### 3.3.2. Precision in Practice: Utilizing Targeted Methods for Geriatric Assessment

In addition, many nurses stated that they rely on the use of standardized measures to guide their practice, such as ADL (Activities of Daily Living), MMSE (Mini-Mental State Examination), and depression assessment scales, to ensure that the assessments are thorough and capture key dimensions of geriatric health.

*“ADL, MMSE, 4AT, 2 depression screens, GDS15, FROP, MNA… These tools help us evaluate each dimension consistently.”*

(FIN_RN2)

*“I feel confident administering the MMSE memory test and interpreting the results to assess where people are at, and yes, that’s the main tool I use.”*

(ICL_RN6)

### 3.4. Theme 2. Balancing Consistency and Flexibility in Assessment Approaches

The second theme included four subthemes: *(a) adapting assessment frequency for dynamic needs, (b) enhancing clarity and consistency in documentation practices, (c) collaborative care in practice: a team-based approach, and (d) proactive risk identification in the early phases*.

Nurses argued that consistency and flexibility in assessing older people are fundamental to providing suitable treatment and care. Consistency ensures reliable monitoring of changes in their health, while flexibility allows adaptation to their changing needs and situations. This balance enhances the effectiveness of care, ensuring the best possible quality of life and early detection of potential health problems.

#### 3.4.1. Adapting Assessment Frequency for Dynamic Needs

Nurses highlighted the importance of awareness and the ability to adapt the assessment frequency to patient needs, especially when health conditions change.

*“The frequency is different for different people, once a year is ok, if something happens, then the situation changes, relatives or neighbors wonder about unusual behavior. The more fragile the patient, all treatment procedures should include a geriatric assessment. Everything is done all the time.”*

(FIN_RN4)

*“I usually always follow up maybe after six months or so, then I call the person and ask how things are going.”*

(ICL_RN5)

#### 3.4.2. Enhancing Clarity and Consistency in Documentation Practices

Documentation was also an important aspect that emerged during the focus group. Nurses stated that including photo updates and detailed records is a practice that supports continuity of care and enables a shared understanding among the care team.

*“We also frequently update each patient’s photos in their file for complete documentation of their condition. This assessment helps us provide personalized care and monitor patient progress.”*

(GR_RN4)

*“But the doctors often refer to my notes, they are detailed, and.”*

(ICL_RN5)

#### 3.4.3. Collaborative Care in Practice: A Team-Based Approach

Moreover, nurses emphasized the importance of teamwork. Participants described working alongside doctors, physiotherapists, and social workers, recognizing that a team-based approach allows for more comprehensive evaluations and effective care planning.

*“They assess when the client enters, then there is a nurse assessment, a doctor assessment, and a social worker assessment.”*

(LT_RN4)

*“Relatives, is essential. Relatives are quite a big resource, helpers in care, and sometimes it is very important to have relatives present to provide this care…”*

(LT_RN4)

#### 3.4.4. Proactive Risk Identification in the Early Phases

One important aspect during CGA is risk assessment. According to the nurses’ responses, CGA allowed for the early detection of risks, such as falls, malnutrition, or polypharmacy, which can prevent severe health deterioration.

*“Importance of identifying risks in the initial phase and emphasizing prevention. In the future, I would like to be able to identify and map the risks even before they start to appear, so that we can effectively identify and refer the elderly to treatment earlier.”*

(FIN_RN3)

*“Falls risk assessment, malnutrition risk assessment… These tools help target and address specific risks.”*

(LT_RN3)

### 3.5. Theme 3. Advancing Expertise: Structured Learning in CGA Principles and Tools

This theme is focused on educational needs and consists of five subthemes: *(a) insufficient formal education frameworks, (b) fostering team synergy: training for effective communication, (c) training for managing time and resource limitations, (d) evidence-based validation of CGA’s influence on patient outcomes, and (e)equipped for diversity: training to address varied patient needs*.

According to the participants, a structured training program in CGA is critical to improving the skills of healthcare professionals in managing older patients. Training enhances their ability to fully appreciate the physical, psychological, and social needs of the elderly, promoting the effectiveness of care. In addition, it promotes the collaboration of multiple healthcare professionals and enhances the implementation of individualized and multifaceted strategies to improve patient outcomes.

#### 3.5.1. Insufficient Formal Education Frameworks

Many participants reported a lack of structured training on CGA, relying instead on on-the-job learning. They expressed the need for courses covering CGA principles, techniques, and tools.

*“I am not [trained in CGA] … First, we would need information about what it means and why it is done.”*

(FIN_RN1)

*“And what I also miss in this kind of evaluation is a single tool that is linked to hospitals and also to general practitioners’ practices.”*

(LT_RN5)

#### 3.5.2. Fostering Team Synergy: Training for Effective Communication

Nurses also identified a need for training on interprofessional communication to support collaboration with doctors, social workers, and families in the CGA process.

*“Effective communication with the patient and family is critical to decision-making, while proper documentation of findings ensures continuity of care and collaboration with other health professionals.”*

(GR_RN3)

*“The geriatric assessment is often a multi-professional, holistic task… Understanding everyone’s role is crucial.”*

(FIN_RN3)

#### 3.5.3. Training for Managing Time and Resource Limitations

With limited time and high patient-to-staff ratios, nurses expressed a need for training on managing assessments within resource constraints, including prioritizing key aspects of CGA.

*“It’s a lack of time and staff… We need training on how to assess effectively under these conditions.”*

(LT_RN1)

*“We try to implement regular health checks and update the history in their file… This documentation allows us to monitor progress.”*

(GR_RN5)

#### 3.5.4. Evidence-Based Validation of CGA’s Influence on Patient Outcomes

Nurses also underscored the importance of data and training to gain a full picture of how CGA affects patient outcomes, quality of life, and healthcare costs. Such insights are essential to providing appropriate care and justifying necessary resource allocation.

*“Verification of efficiency and cost-effectiveness is important… We need data to support decision-makers.”*

(FIN_RN3)

Then, providing training on how CGA reduces hospitalizations and supports at-home care was highlighted as a priority. This education helps ensure that both nurses and policymakers recognize the importance of the intervention in delivering appropriate care.

*“The assessment helps identify problems… It enhances quality of care and quality of life for the elderly.”*

(GR_RN3)

#### 3.5.5. Equipped for Diversity: Training to Address Varied Patient Needs

Given the multicultural settings in which many nurses work, training on cultural competence and sensitivity in elderly care was identified as a gap. Understanding diverse values, preferences, and communication styles was seen as important for effective CGA.

*“Understanding the individual needs and preferences of seniors is critical.”*

(GR_RN6)

*“Allows for personalized care, which is important in promoting their health and well-being.”*

(GR_RN3)

### 3.6. Cross-Cohort Variations in Focus Group Discussions

Although the thematic analysis identified shared global themes across all participants, distinct variations in emphasis were observed between the individual national cohorts during the focus group discussions. The Finnish cohort placed a pronounced emphasis on clinical precision, the heavy use of standardized assessment instruments (such as the MMSE, 4AT, and MNA), and role adjustments linked to clinical task-shifting. In contrast, the Icelandic nurses focused on the long-term continuity of care, autonomous nursing follow-ups, and structured communication frameworks with physicians. Within the Baltic region, the Estonian cohort highlighted the pressing need for unified digital platforms linking general practices to hospitals, while the Latvian cohort heavily emphasized the active integration and utilization of informal family caregivers as essential structural resources within the care team. Finally, the Greek nurses uniquely emphasized meticulous physical file documentation, patient privacy tracking, and population-level community health campaigns.

## 4. Discussion

This study explored primary health care nurses’ experiences and educational needs related to the implementation of CGA across five European countries. The findings indicate that while nurses recognize the value of CGA and actively apply many of its components in practice, there remains a substantial need for structured education and clearer frameworks to support consistent and effective implementation. The results highlight both existing good practices and persistent systemic gaps that affect the quality and sustainability of geriatric care in PHC.

Participants described the routine use of various assessment tools, such as the ADL and MMSE, to support a comprehensive evaluation of older adults. This aligns with previous research identifying clinical assessment and diagnostic competence as core components of PHC nursing practice [14]. Assessing functional status, cognition, nutrition, and psychological well-being enables early identification of risks and supports individualized care planning. The widespread use of validated tools reinforces their central role in ensuring accuracy, reliability, and comparability in CGA implementation [23,24,25]. In addition, participants highlighted the increasing use of digital tools and electronic documentation systems, which may facilitate more efficient and systematic assessments and improve continuity of care [26].

Interdisciplinary collaboration emerged as a central pillar of effective CGA. Nurses emphasized the importance of close cooperation with physicians, physiotherapists, social workers, and other professionals to develop individualized care plans. This form of “team synergy” goes beyond information exchange and reflects shared decision-making aimed at improving patient outcomes. Consistent with previous research, effective interprofessional collaboration is associated with improved care quality, reduced medical errors, and better patient safety outcomes [27,28]. Participants also stressed the need for targeted training in communication and teamwork skills, which are essential for sustaining collaborative practice and ensuring continuity of care in PHC settings [29].

Collaboration was also extended to family caregivers, who were recognized as a crucial resource in the care of older adults. Participants emphasized that family members frequently support medication management, symptom monitoring, transportation, and emotional well-being. Involving caregivers in the CGA process facilitates more accurate information gathering and supports shared understanding of care goals. At the same time, caregiver engagement provides opportunities for guidance and education, thereby strengthening their capacity to support older persons effectively [11,30,31]. These findings reinforce the importance of viewing CGA as a collaborative process that integrates professional and informal care networks.

Another prominent theme concerned the role of documentation in supporting continuity and quality of care. Participants highlighted the importance of detailed, up-to-date records, including visual documentation, to track changes in health status and facilitate information sharing among team members. High-quality documentation enables timely identification of subtle clinical changes, which is particularly important in older adults where minor deviations may signal serious deterioration [32,33]. In this context, documentation functions not merely as an administrative task but as an active clinical tool that supports decision-making, patient safety, and coordinated care delivery.

Resource constraints, particularly time pressure and staffing limitations, were identified as significant barriers to effective CGA implementation. Participants emphasized the need for training in prioritization and time-management strategies to ensure that essential components of CGA can be performed even under constrained conditions. Previous research supports the association between adequate nurse staffing and improved patient outcomes, including reduced complications and mortality [34,35]. Conversely, insufficient resources contribute to occupational stress and burnout, further compromising care quality [36]. Integrating resource-management competencies into nursing education may, therefore, enhance both care effectiveness and workforce sustainability.

Cultural competence also emerged as an important area for development. Increasing cultural diversity in many European societies requires nurses to adapt CGA processes to different linguistic, cultural, and belief systems. Insufficient training in cultural sensitivity may lead to misunderstandings, reduced adherence, or inequities in care delivery [37]. Developing cultural humility and communication skills has been shown to improve patient safety and engagement [38]. Consequently, CGA education programs should incorporate intercultural competencies to ensure equitable and person-centered assessment practices.

A particularly important finding of this study is the reported lack of structured and standardized CGA education within nursing curricula. Despite the recognized importance of CGA, geriatric content is often fragmented, inconsistently delivered, or embedded within broader nursing courses rather than treated as a distinct competency area [39,40,41,42,43]. International evidence highlights wide variability in curriculum content, duration, and pedagogical approaches [40,44], limiting the preparedness of nurses to conduct CGA independently. Recent work has identified six core competency domains for CGA in PHC nursing—clinical assessment and diagnostics, care coordination, professional and interpersonal skills, environmental and systemic competencies, technical skills, and quality improvement—which provide a useful foundation for curriculum development [14]. The themes identified in the present study align closely with these domains, supporting their relevance and applicability across different European contexts.

### 4.1. Cross-Contextual Variations and Systemic Drivers

When interpreting the findings, it is important to note that the role and scope of PHC nursing vary across the five participating European countries, reflecting their distinct health system architectures. These structural and operational variations directly influence how CGA is interpreted, prioritized, and operationalized in daily clinical practice.

In Finland, PHC nursing is structurally designed around health promotion and preventative care, utilizing intentional interprofessional collaboration and a progressive task-shifting model from physicians to nursing staff to optimize population health outcomes. The Finnish infrastructure provides a seamless, life-course-integrated network of public health services spanning maternal, child, school, occupational, and geriatric care, all aligned with national age-specific health priorities [45]. In the focus groups, the Finnish emphasis on standardized tool management (such as the MMSE, 4AT, and MNA) and task-shifting clarity directly reflected this highly institutionalized, municipal framework. However, the data indicate that such heavily mandated data collection can paradoxically trigger clinical tool-fatigue among frontline clinicians.

Conversely, in Iceland, primary care policy similarly emphasizes bringing nurses to the forefront of PHC by expanding task-sharing and independent clinical responsibilities, backed by dedicated postgraduate primary care nursing training pathways [46]. The core focus of Icelandic participants on long-term care continuity and high clinical independence aligns perfectly with these systematic, decade-long national policy reforms aimed at expanding autonomous clinical boundaries. In the Baltic context, the specific challenges voiced by the cohorts reflect distinct regional system dynamics. In Estonia, PHC nursing is deeply integrated into family medicine practices where family nurses operate independently alongside general practitioners to manage acute and chronic demands, and as of 2022, are authorized to issue sick leave certificates and specific medications, provided they have undergone the necessary training [47]. Consequently, the Estonian focus group’s demand for integrated digital tools stems directly from this independent operational framework, which requires seamless cross-sectoral communication to manage heavy chronic workloads.

In Latvia, PHC nurses are undergoing transitional reforms, bridging older polyclinic infrastructures and private family practices, while shifting focus from traditional administrative or assistant roles toward independent clinical health promotion [48]. The prominent emphasis among Latvian participants on utilizing informal family networks to support multi-professional evaluations highlights this transitional state; as the infrastructure shifts toward multidisciplinary family practices, nurses frequently leverage family relatives to bridge public resource gaps in community home care.

Lastly, in Greece, primary care operates under a hybrid framework where PHC nurses are situated within public municipal health centers or local healthcare units. Their role heavily involves disease prevention, community health education, vaccination programs, and public hygiene, alongside collaborative multidisciplinary clinical support [49]. The Greek nurses’ distinct focus on meticulous physical file documentation, patient privacy tracking, and local file updates reflects this specific architecture. Operating within these local units, Greek PHC nurses must navigate high patient volumes and severe resource constraints, forcing them to maximize localized documentation strategies to sustain care continuity.

Across all five nations, unlike some community models globally that rely on volunteer networks, these professionals are university-educated, registered clinicians embedded directly within state-regulated healthcare infrastructures. While nurses in highly autonomous systems (such as Finland and Iceland) may require advanced training focused on clinical leadership and independent decision-making within CGA, those in transitional or hybrid systems (such as Latvia and Greece) require educational frameworks that strengthen foundational competency, time management, and role definition within multidisciplinary teams. Ultimately, these differences demonstrate that while all these nations face the universal pressure of an aging population, a single consolidated geriatric curriculum is unsustainable. Effective CGA implementation across Europe requires a flexible educational framework that establishes a unified standard of core geriatric competencies while respecting and adapting to the specific national scopes of practice.

### 4.2. Strengths and Limitations

Several limitations should be acknowledged. The qualitative design and relatively small sample size per national cohort (29 participants in total across five countries) limit statistical generalizability. However, in qualitative methodology, and specifically regarding focus group research, the literature systematically indicates that small, cohesive groups of five to eight participants are optimal to facilitate interactive dialogue, prevent group fragmentation, and ensure that all members can provide in-depth professional insights [50]. Furthermore, because the overarching objective of this international consortium was to map shared core competencies to develop a unified European curriculum rather than to perform a localized comparative epidemiological analysis, the total sample of 29 highly experienced primary care clinicians provided sufficient thematic saturation and information power to justify the reliability and transferability of our analytical conclusions.

The gender imbalance, with a predominance of female participants, may restrict the representation of male nurses’ perspectives. However, rather than indicating a selection bias, this demographic distribution heavily reflects the historical and contemporary reality of the nursing workforce across the participating European regions, where female professional dominance remains overwhelmingly the norm [51]. While gender-specific clinical reasoning styles cannot be fully isolated within this framework, the focus on core pedagogical developments and universal geriatric competencies suggests that this imbalance did not fundamentally distort the identification of main educational needs.

Furthermore, variations in the participants’ professional longevity (ranging from 2.5 to 40 years) and baseline geriatric education could be perceived as an interpretive variable. In reflexive qualitative inquiry, however, this heterogeneity is viewed not as a methodological bias but as a significant analytical asset. Including both early-career nurses, who offer critical insights into recent transition-to-practice educational gaps, and highly seasoned clinicians establishes a multi-layered spectrum of perspectives. Similarly, while prior exposure to specialized geriatric training inherently heightens a clinician’s sensitivity toward educational deficits, this critical reflexivity directly serves the pragmatic goals of this study, which aims to map out realistic, comprehensive curriculum benchmarks for future primary care nursing education.

Data were self-reported and may be influenced by recall or social desirability bias. Furthermore, conducting focus groups in multiple languages introduces the possibility of subtle meaning loss during transcription and translation. Nevertheless, the inclusion of experienced nurses from diverse healthcare systems strengthens the credibility of the findings. The multicountry design and use of thematic analysis enabled the identification of shared challenges and educational needs across contexts, enhancing the insight into this matter.

Overall, this study provides valuable insights into how PHC nurses perceive and implement CGA in everyday practice. The findings highlight the need for structured, competency-based education that integrates clinical assessment skills, interdisciplinary collaboration, documentation, cultural competence, and resource management. Strengthening such educational frameworks may enhance the quality, consistency, and sustainability of CGA in PHC. In an increasingly aging European population, investing in nursing education and supportive systems is essential to ensure high-quality, person-centered geriatric care.

## 5. Conclusions

This international study highlights that PHC professionals view CGA as a holistic process integrating physical, psychological, and social domains of care. Key elements identified include effective team collaboration, adequate resources, and the need for structured education to support consistent CGA implementation. These findings provide a foundation for developing targeted geriatric nursing curricula that address the multidimensional learning needs of adult learners.

Educational approaches should move beyond theory to include structured training in standardized assessment tools and interprofessional communication, enabling nurses to manage complex clinical situations in everyday practice. In addition, evidence demonstrating the impact of CGA on outcomes such as reduced hospitalizations and prevention of functional decline is essential to inform policy decisions and justify investments in staffing and educational resources. Strengthening these areas may support the delivery of high-quality, cost-effective, and person-centered geriatric care.

## Figures and Tables

**Table 1:** Participants’ characteristics.

Characteristic	Category	*n* (%)	Mean (Range)
Gender	Female	27 (93.1%)	
	Male	2 (6.9%)	
Country of Practice	Finland	6 (20.7%)	
	Estonia	5 (17.2%)	
	Latvia	5 (17.2%)	
	Greece	6 (20.7%)	
	Iceland	7 (24.1%)	
Education Level	MSc	9 (31.0%)	
	BSc/Cert	15 (51.7%)	
	Vocational/RN Diploma	5 (17.2%)	
Years of Experience			16.3 (2.5–40)
Age (Years)	Recorded	24 (82.8%)	47.6 (24–66)
	Not available	5 (17.2%)	

BSc: Bachelor’s Degree, MSc: Master’s Degree, RN: Registered Nurse.

**Table 2:** Thematic analysis results.

Themes	1. Embracing Complexity: A Holistic Framework for Comprehensive Assessment	2. Balancing Consistency and Flexibility in Assessment Approaches	3. Advancing Expertise: Structured Learning in CGA Principles and Tools
Subthemes	1.1 Intersecting domains: Physical, psychological, and social well-being	2.1 Adapting assessment frequency for dynamic needs	3.1 Insufficient formal education frameworks
	1.2 Precision in practice: Utilizing targeted methods for geriatric assessment	2.2 Enhancing clarity and consistency in documentation practices	3.2 Fostering team synergy: Training for effective communication
		2.3. Collaborative care in practice: A team-based approach	3.3 Training for managing time and resource limitations
		2.4 Proactive risk identification in the early phases	3.4 Evidence-based validation of CGA’s influence on patient outcomes
			3.5 Equipped for diversity: Training to address varied patient needs

CGA: Comprehensive Geriatric Assessment.

## Data Availability

The data presented in this study are available from the corresponding author on request due to ethical restrictions.

## Supplementary Materials

The following supporting information can be downloaded at: https://www.mdpi.com/article/10.3390/geriatrics11030073/s1, Table S1: Revised COREQ Checklist.
