# Supplementary material for: From Complexity to Competency: International Nursing Perspectives on Comprehensive Geriatric Assessment
Source: Geriatrics (Basel). 2026 Jun 17;11(3):73. doi: 10.3390/geriatrics11030073 (PMC13299193; doi:10.3390/geriatrics11030073)
Supplement: Supplementary file 1 [file geriatrics-11-00073-s001.zip › geriatrics-4303008-supplementary.pdf]

Revised COREQ Checklist

**No.**	**Item**		**Page / Response**
**Domain 1**	**Research team & reflexivity**		
1	Interviewer/facilitator	Which author/s conducted the interview or focus group?	Page 5; Bilingual local research team members from each partner nation consortium.
2	Credentials	What were the researcher's credentials?	Page 1; Affiliated co-authors hold university nursing faculty positions, PhDs, MScs, and RN certifications.
3	Occupation	What was their occupation at the time of the study?	Page 1 & 5; Nursing educators, clinical researchers, and public health experts.
4	Gender	Was the researcher male or female?	Female and Male representation across the international research consortium.
5	Experience and training	What experience or training did the researcher have?	Page 1 & 5; Experienced international consortium in PHC, geriatric care, and qualitative research methods.
6	Relationship established	Was a relationship established prior to study commencement?	Researchers had no reported prior professional or ongoing clinical relationships with participants prior to recruitment.
7	Participant knowledge	What did the participants know about the researcher?	Minimal; participants were informed of the overarching goals of the Erasmus+ framework project.
8	Interviewer characteristics	What characteristics were reported about the interviewer?	Pages 5-6; The focus group facilitators were bilingual nursing faculty and clinical researchers embedded within their respective national healthcare

			systems. To acknowledge, manage, and mitigate potential professional subjectivities, regional health system assumptions, and interpretive biases, the international research team utilized reflexive journals and structured, cross-country peer-debriefing sessions throughout the data analysis and theme development phases.
**Domain 2**	**Study design**		
9	Methodological orientation	What methodological orientation underpinned the study?	Pages 5-6; Reflexive Thematic Analysis (Braun & Clarke framework).
10	Sampling	How were participants selected?	Page 3; Purposive sampling through the international consortium network across 5 European nations.
11	Method of approach	How were participants approached?	Page 3; Approached via institutional/consortium outreach networks within partner countries. Page 6-7; Participants were approached via institutional networks and provided with formal participant information sheets. Written informed consent was explicitly obtained from all participating nurses prior to the commencement of the

			focus groups.
12	Sample size	How many participants were in the study?	Page 1 & 3; 29 primary health care nurses.
13	Non-participation	How many people refused or dropped out? Reasons?	Page 3; Zero dropouts or refusals reported; all selected members completed focus group sessions.
14	Setting of data collection	Where was the data collected?	Page 3; Face-to-face focus group sessions within each respective participating country.
15	Presence of non-participants	Was anyone else present besides participants/researchers ?	Page 3; Only the clinical participants and the local bilingual research facilitators were present.
16	Description of sample	What are the important characteristics of the sample?	Page 4, Section 3.1 & Table 1; Detailed demographics (gender, country, education, clinical experience) were collected via a brief, pre-interview demographic questionnaire completed by participants prior to the sessions.
17	Interview guide*	Were guides provided? Was it pilot tested?	Page 4, Section 2.2; A standardized, semi-structured focus group interview guide consisting of 14 specific clinical and educational questions was utilized across all five countries to ensure structural consistency.<br><br>While a formal external pilot study was not conducted, the interview guide was subjected to a rigorous internal pilot

			evaluation and peer-review process by the international expert consortium co-authors to ensure cross-linguistic equivalence, cultural adaptability, and conceptual clarity prior to deployment.
18	Repeat interviews	Were repeat interviews carried out?	No repeat interviews were conducted; single-session focus groups.
19	Audio/visual recording	Did the research use audio or visual recording?	Page 5; Audio-recorded and transcribed verbatim.
20	Field notes	Were field notes made during/after?	Page 5,6; Reflexive journaling was utilized during data analysis.
21	Duration	What was the duration of the focus groups?	Page 4; Sessions lasted between 92 and 117 minutes.
22	Data saturation	Was data saturation discussed?	Page 11; Section 4.2 states that 29 highly experienced clinicians provided sufficient thematic saturation.
23	Transcripts returned	Were transcripts returned to participants?	No transcripts were returned to participants for correction.
**Domain 3**	**Analysis and findings**		
24	Number of data coders	How many data coders coded the data?	Page 5-6, Section 2.3 & Section 2.4; Two data coders independently coded a representative subset of the qualitative transcripts. The coders then met to compare codes, resolve interpretative variations, and establish a single, unified coding framework via consensus, which was

			applied across the entire dataset to ensure rigor and credibility.
25	Description of coding tree	Did authors provide a description of the coding tree?	Page 8; Table 2 outlines the structural architecture of themes and subthemes.
26	Derivation of themes	Were themes identified in advance or derived?	Page 5; Derived inductively through reflexive thematic analysis of the empirical dataset.
27	Software	What software was used to manage the data?	Not applicable; data were managed via manual qualitative tracking and multi-country team synthesis.
28	Participant checking	Did participants provide feedback on findings?	No participant checking or member checking was performed on final themes.
29	Quotations presented	Were participant quotations presented?	Pages 9-11; Illustrative quotes provided for subthemes, identified by country and participant code (e.g., FIN_RN1, ICL_RN6).
30	Data/findings consistency	Was there consistency between data and findings?	Yes; findings are directly grounded in and supported by translated verbatim participant quotes.
31	Clarity of major themes	Were major themes clearly presented?	Yes; three major themes are clearly articulated in the narrative and in Table 2.
32	Clarity of minor themes	Is there a discussion of minor themes/diverse cases?	Pages 5, 12, & Section 2.4; Yes. Confirmability and transferability were strengthened by explicitly presenting localized, cross-cohort national variations (Section 3.6) alongside the three global themes.

			The text details how distinct health system architectures (e.g., tool-fatigue in Finland, informal caregiver reliance in Latvia) shaped unique clinical nuances among the cohorts.

*Focus Group Interview Guide: the exact core questions utilized during the focus groups are listed below:

1. How long have you been working as a nurse in primary health care/social and health care?
2. What has been your experience with older adults in your practice? geriatric assessment?
3. Have you received education on how to carry out comprehensive geriatric assessment and what kind of education?
4. Could you describe what and how you understand by comprehensive geriatric assessment? What experiences do you have with geriatric assessment?
5. How often do you have to use geriatric assessment in your work?
6. How do you perform the health and evaluation of the elderly?
7. Describe the main areas that arise specifically in the nurse's work in this geriatric assessment
8. What tools do you use when you do a geriatric assessment? e.g. work tools, colleague support, mentors
9. In your opinion, how effective is the geriatric assessment in improving the care of older adults?
10. What challenges do you think there could be in this geriatric assessment?
11. Can you describe a situation where you felt certain, successful or uncertain about the matter?
12. What areas do you feel you need more education in regarding elderly assessment?
13. How do you currently update your knowledge and skills in caring in geriatric care?
14. What are your ideas for developing education (contents, methods and evaluation) of comprehensive geriatric assessment?
